# Tomas A. Salerno: Visionary, Innovator, Friend

**DOI:** 10.21470/1678-9741-2022-0950

**Published:** 2022

**Authors:** Ricardo de Carvalho Lima

**Affiliations:** 1 Faculdade de Ciências Médicas, Universidade de Pernambuco (UPE), Recife, Pernambuco, Brazil; 2 Pronto-Socorro Cardiológico Universitário de Pernambuco (PROCAPE), Universidade de Pernambuco (UPE), Recife, Pernambuco, Brazil

“*The future is now, and I wish I was born today” Tomas A. Salerno*

## INTRODUCTION - BIRTH AND UPBRINGING

Tomas A. Salerno - BSc, MSc, FRCS (C), FACS, FCCP (surgery), Professor of Cardiothoracic Surgery, and DeWitt Daughtry Endowed Chair in Cardiothoracic Surgery at the University of Miami Miller School of Medicine - has been recognized internationally for over 40 years as one of the most prominent and innovative academic cardiac surgeons in the field.

**Fig. 1:**
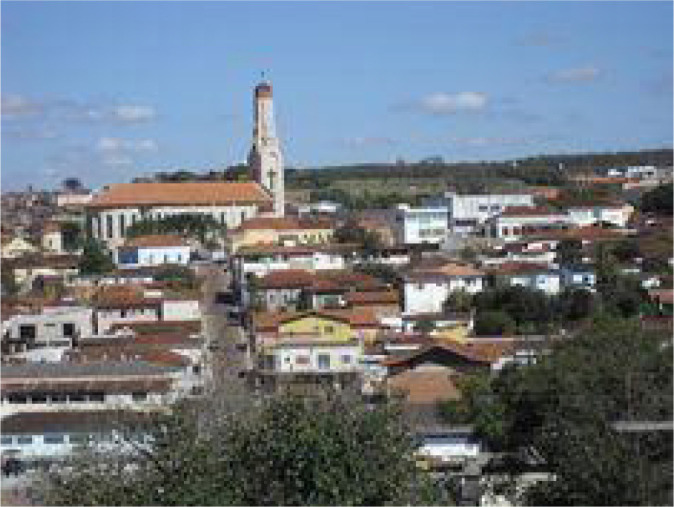
City of Cássia, countryside of Minas Gerais state, Brazil.

**Fig. 2:**
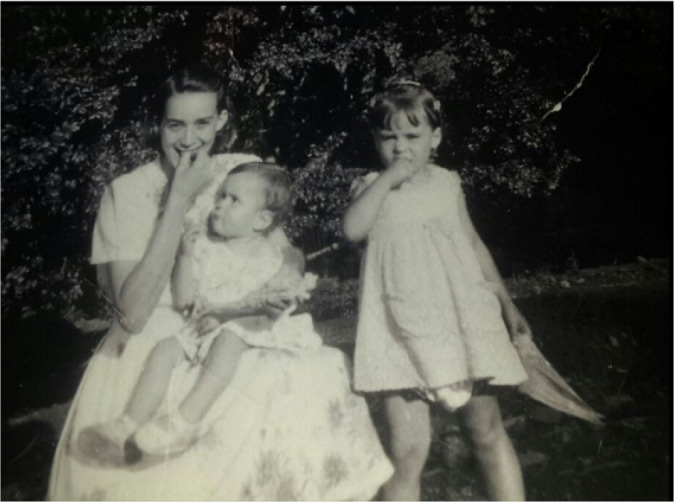
Tomas, his mother Silvéria, and his sister Telma.

**Fig. 3:**
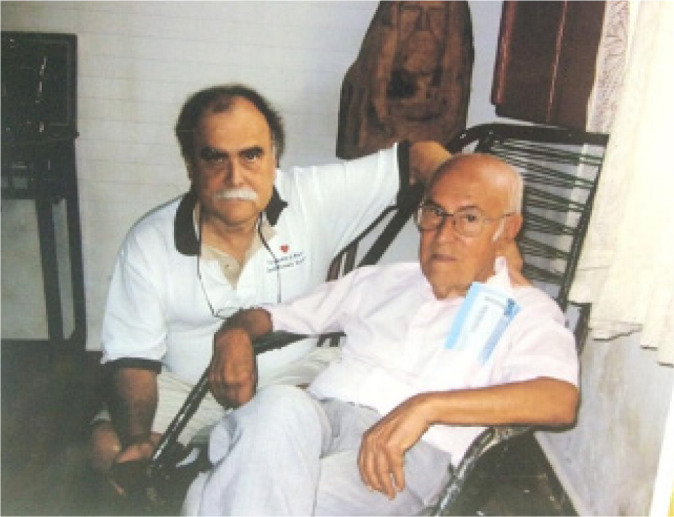
Tomas with his father José in the city of Cássia, Brazil.

Tomas and his sister Telma were born into a modest family, in Cássia (Figure 1), a small town in the state of Minas Gerais (Brazil). His father, José Salerno, was self-employed, and his mother, Silvéria F. Salerno, was a housewife, dedicated to the care of their home and family (Figures 2 and 3). He attended the first part of high school in his hometown, at São Gabriel School, and the second part in the city of São Paulo, where he worked through his schooling - teaching English during the day to make money and attending school at night.

**Figure:**
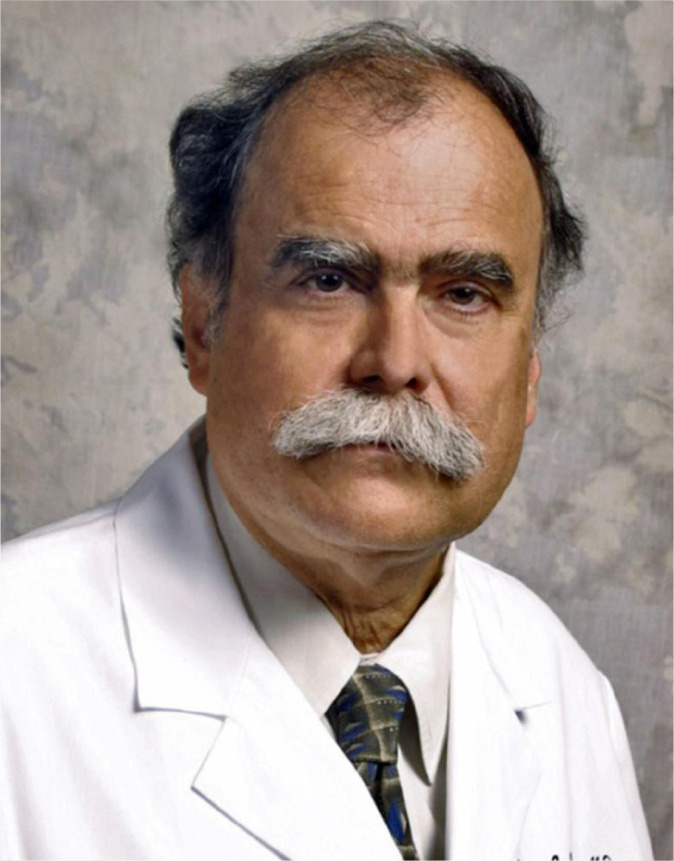
*Tomas A. Salerno*

## EDUCATION AND SURGICAL TRAINING

**Fig. 4:**
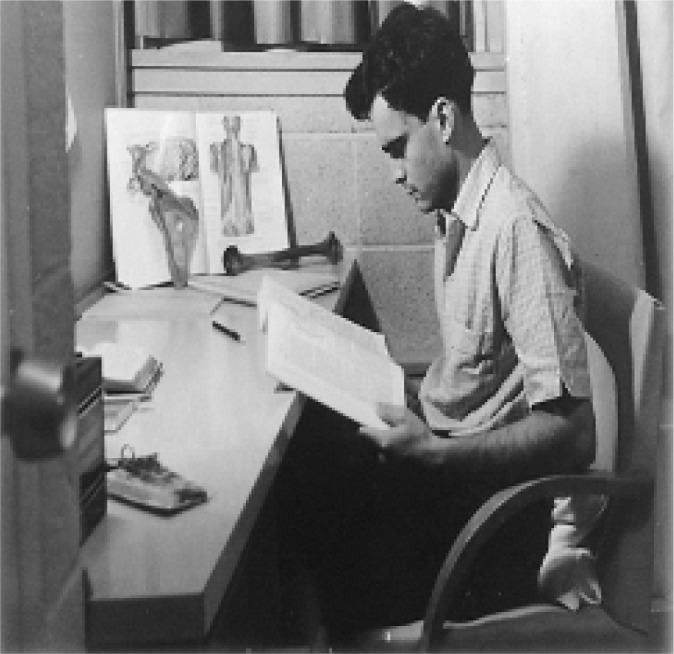
Tomas as a medical student at McGill University.

**Fig. 5:**
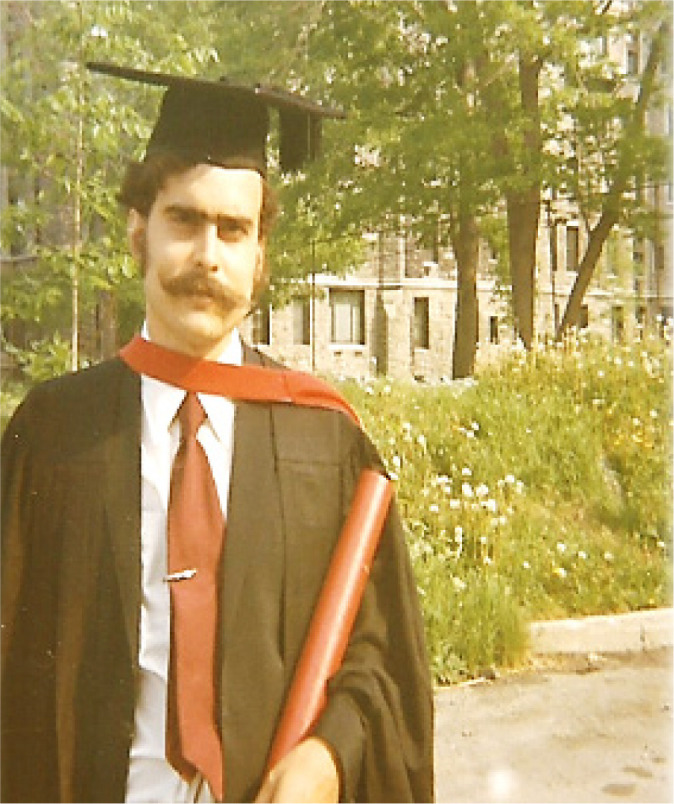
Tomas obtained BSc honors in Biochemistry at McGill University.

In 1963, Tomas went to Brattleboro, Vermont (United States of America [USA]), to teach in the Peace Corps’ volunteer training and decided that the USA was the country he wanted to live in. Then, he went to New York and obtained a full scholarship from the Leopold Schepp Foundation to study undergraduate and graduate courses at McGill University, in Canada, where he obtained his MD (Figure 4), trained in general surgery and cardiothoracic surgery, and obtained a Masters’ degree (Figure 5).

## ACADEMIC SURGICAL CAREER CURRICULUM VITAE HIGHLIGHTS

**Fig. 6:**
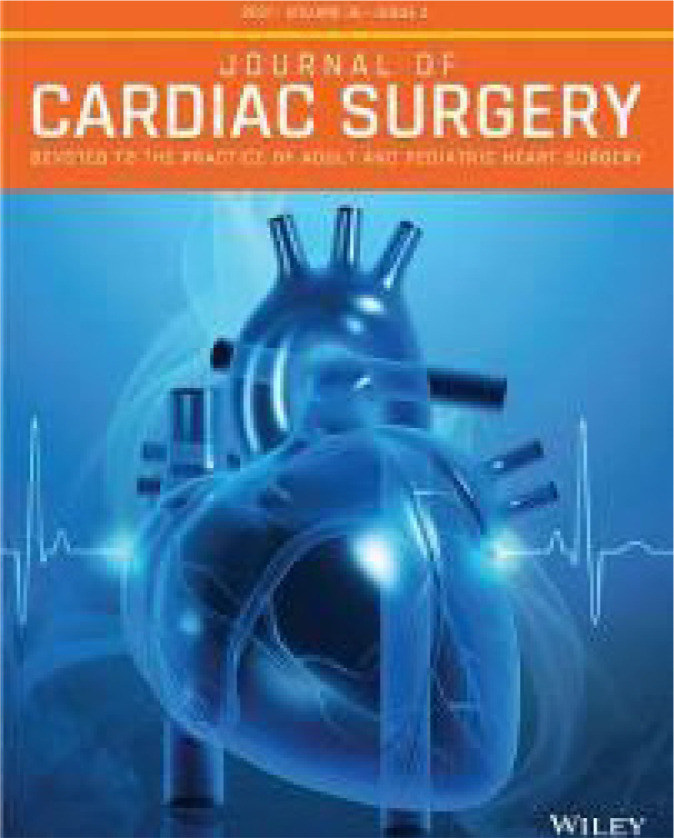
Dr. Salerno is currently the Editor-in-Chief of the Journal of Cardiac Surgery.

Dr. Salerno has held academic leadership positions as Chief of Cardiothoracic Surgery at McGill University, University of Toronto (Canada), State University of New York at Buffalo, and Miami University. He is board certified in Cardiothoracic Surgery in Canada and USA. And he is member of all relevant societies in the specialty of Cardiothoracic Surgery, including the Brazilian Society of Cardiovascular Surgery. After a national and international search, Dr. Salerno was recently appointed Editor-in-Chief of the Journal of Cardiac Surgery, a prestigious and leading peer-review journal in the USA (journal impact factor 1.62) (Figure 6).

## CANADA YEARS (1973 - 1992)

**Fig. 7:**
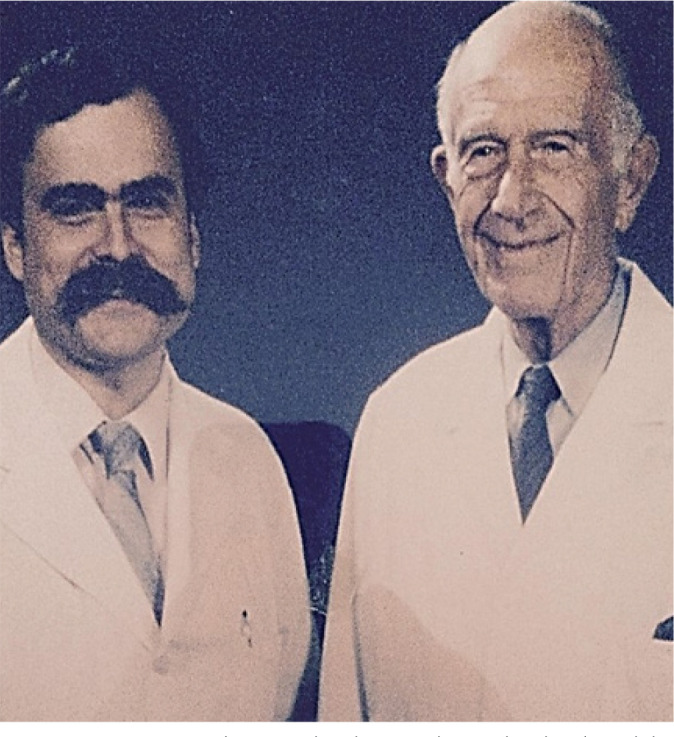
Drs. Tomas Salerno and Arthur Vineberg who developed the procedure of direct implantation of the internal mammary artery into the left ventricle for the relief of myocardial ischemia.

Tomas graduated in Medicine from McGill University and worked as a student with Arthur Vineberg (Figure 7). He began his medical course and academic career in Quebec, as an Anatomy Instructor and Research Fellow in Cardiovascular and Thoracic Surgery at McGill University, Montreal (Quebec), from July 1973 to June 1974. From August 1977 to September 1982, he held the position of Assistant Professor and then Associate Professor in the Department of Surgery at Queen’s University, Kingston (Ontario). In October 1982, he went back to McGill University as Associate Professor of Surgery, where he stayed for a year.

*“If I have seen further, it is by standing on the shoulders of giants*”

Sir Isaac Newton, 1675

**Fig. 8:**
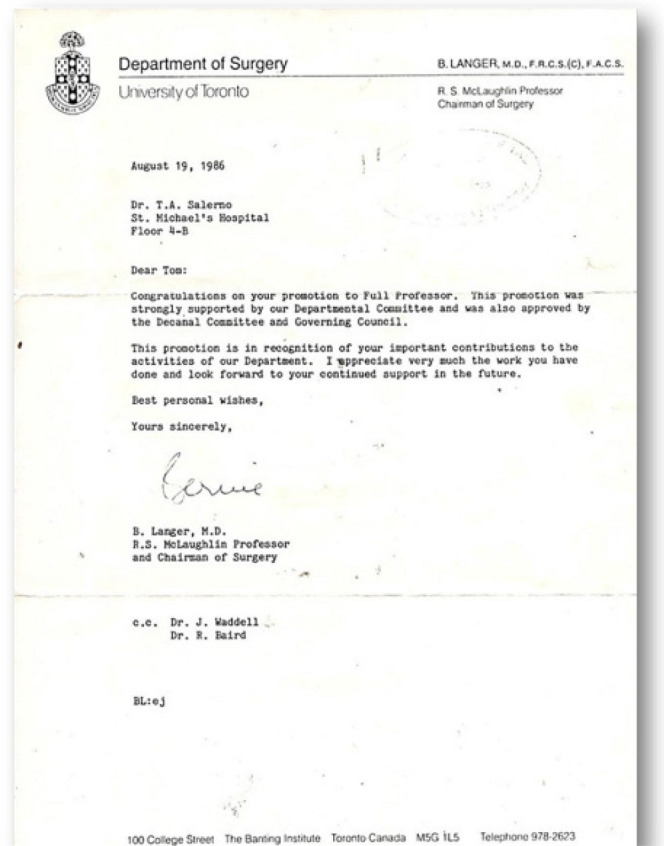
Letter from Dr. Bernard Langer to Dr. Tomas A. Salerno communicating the position of Full Professor of Cardiovascular Surgery.

In 1986, Dr. Salerno was promoted to the position of Full Professor in the Department of Surgery at the University of Toronto (Figure 8) supported by Professor Bernard Langer, who was a Canadian surgeon and educator, and, in his honor, the University of Toronto has established the Bernard and Ryna Langer Chair in General Surgery, the Department of Surgery Langer Surgeon Scientist Award, and the Bernard Langer Annual Lecture in Health Sciences. Also, the Canadian Association of General Surgeons has a Langer Lecture at its annual meeting. In 2002, Langer was named an Officer in the Order of Canada and in 2015, he was inducted into the Canadian Medical Hall of Fame.

The content of the letter from Professor Bernard Langer emphasizes Dr. Salerno’s work and recognition from his peers - *“This promotion was strongly supported by our Departmental Committee and also approved by the Decanal Committee and Governing Council”*.

**Fig. 9:**
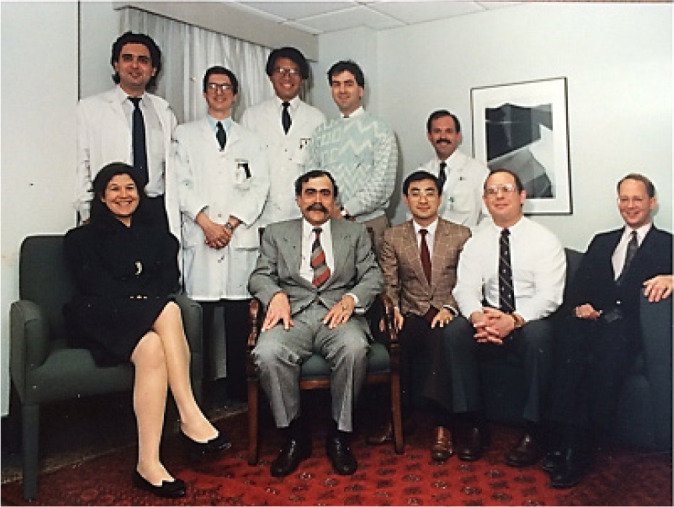
Tomas Salerno with graduating fellows from the University of Toronto in the 1980’s.

As Full Professor of the University of Toronto, Dr. Salerno begins a new phase of his academic life, being Associate Director of the Institute of Medical Science, Professor of Surgery, and Coordinator of the Cardiovascular Section, Clinical Science Division and University Chair Division of Cardiovascular Surgery, Department of Surgery, University of Toronto, until 1992 (Figure 9).

## BUFALLO YEARS (1992 - 1999)

In the year of 1992, Dr. Salerno left the University of Toronto (Canada), moved to Buffalo city, New York (USA), and began his work as Professor of Surgery, Chief of the Division of Cardiothoracic Surgery, State University of New York at Buffalo, where he remained until 1999. During this period, he created an *International Center for Cardiac Surgery*. This center was developed to promote education worldwide in the field of Cardiothoracic Surgery, allowing physicians to go to Buffalo to share their experiences, learn new techniques, teach the students, and participate in an academic environment. This has been an important program, and over 100 physicians from all over the world have participated in this venture in Buffalo. Also, opportunities have been created for students to participate in this program and improve their education in Buffalo.

## MIAMI YEARS (1999 - )

In 1999, Dr. Salerno joined the staff of Miami University being “Highly Recommended”. Very quickly, he became a known and most respected faculty member of this University. He served the University as Chair of the Faculty Senate (the University of Miami’s highest position) from 2014 to 2019, and for many years before, as member of the Faculty Senate and second and first Vice-Chair, despite the fact that he, at the same time, was carrying out a heavy clinical load as a heart surgeon, continued to teach/train fellows, residents, and medical students, and maintained a very productive academic career in research, publications, presentations, and scholarly activities.

**Fig. 10:**
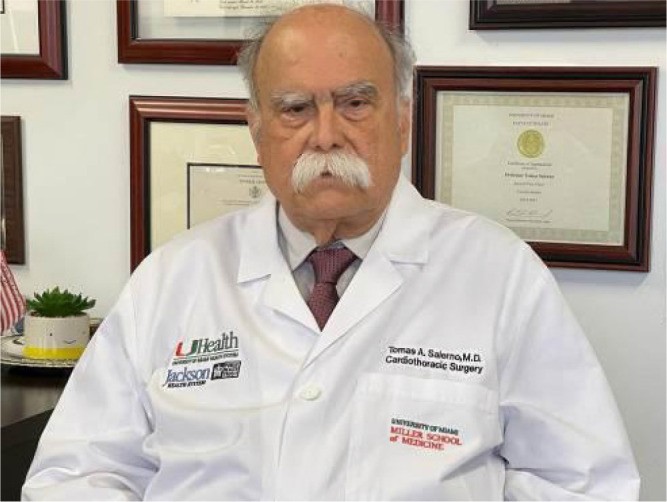
Dr. Tomas A. Salerno in his office at Jackson Memorial Hospital.

Dr. Salerno became one of the most popular Chair of the Faculty Senate, opening communication between the Administration and the Faculty Senate, forging relationships between the various governing bodies of the University, and leading to discussion, collaborative projects, and the commonality of a shared mission. He has shown his strength at getting people together, providing a venue for administrators, board members, and Faculty to meet and develop interdisciplinary relationships. As a Chair of the Faculty Senate, he also served as Chair of the General Welfare Committee, member of the Faculty Senate Budget and Compensation Committee (*ex officio*), member of the Promotions Committee for the Department of Surgery Miller School of Medicine, and as Vice-Chair for the Department of Surgery, Faculty Mentoring, and Development (Figure 10).

## BACKGROUND RESEARCH - BIOGRAPHY AND ACHIEVEMENTS

The contributions made by Dr. Salerno in the field of cardiac surgery are legendary, and they are all based on scientific research, innovative ideas, carefully executed trials, and long-standing support for research from the USA’s National Research Council and industry. Dr. Salerno published, presented, lectured, and taught extensively, as documented in his curriculum vitae. Within his contributions, some can be prioritized:

### Warm Heart Surgery

Cardiac surgery, based on the work of Dr. Bigelow from the University of Toronto, was hypothermia dependent. The so-called “ice age” came about due to observation that cold decreases metabolism and oxygen consumption, and Dr. Bigelow pioneered and made his name on this contribution.

Nowadays, cardiac surgery is carried out under conditions of some degree of hypothermia in order to slow the heartbeat and it is associated with cold cardioplegia, but there still are risks of ischemia in high-risk patients and for high-risk procedures. “Warm Heart Surgery” provides a highly topical review of this controversial area and presents a balanced view of the advantages and disadvantages of the technique. Dr. Salerno was already interested in cardioplegia, leading to warm the temperature of the patient and the heart with excellent results. He became an expert on myocardial protection, particularly warm heart surgery, and lectured worldwide on this topic. All this was based on scientific work on pigs in his laboratory. His published textbook is a classic on this topic^[1]^.

### Atrial Natriuretic Factor (or ANF)

While experimenting on pig hearts in Kingston (Ontario), Dr. Salerno met a pathologist who was working on pinocytes of the pituitary, Dr. Adolfo DeBold, and asked him if he could do microscopic studies in pig hearts. Dr. DeBold asked Dr. Salerno what were the granules that Palade had identified in the cardiac atria (Palade won the Nobel prize) and postulated that the heart was an endocrine organ. Dr. Salerno suggested that the atria of rats should be pulverized, and the supernatant removed and injected into another rat. That led the rat to produce a large amount of urine. Drs. DeBold and Salerno work on a manuscript to the Journal of Cardiovascular and Thoracic Surgery, which was rejected, but they managed to publish a manuscript with their names - *Natriuretic activity of extracts obtained from hearts of different species and from various rat tissues* - in the Canadian Journal of Physiology and Pharmacology^[2]^. This was the beginning of a long investigative work that led Dr. DeBold to receive the Gairdner Award in Toronto.

### Myocardial Protection

**Fig. 11:**
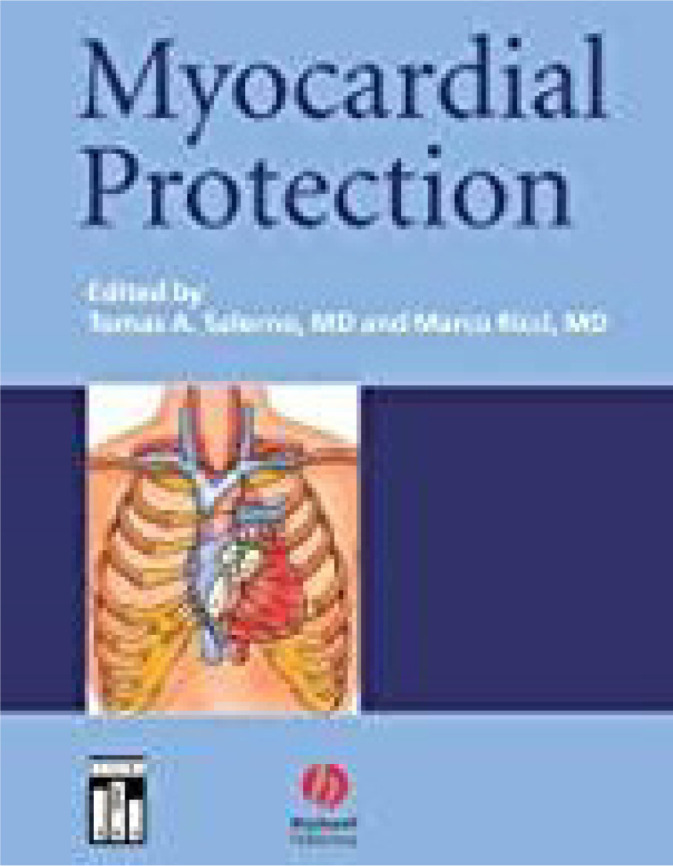
Textbook on cardioplegia and myocardial protection by Drs. Tomas A. Salerno and Marco Ricci.

Through his research work, collaborations with the National Research Council, and the York University in Toronto, Dr. Salerno made important contributions which led to better ways of protecting the heart during cardiac surgery. Working in cooperation with Dr. Gerald Buckberg, a leader in the field, they travelled the world together giving lectures, opposing views, and collaborating in different areas, all leading to important ways of creating solutions to protect the heart. His book on myocardial protection (Figure 11) is one of the few in the field, and Dr. Salerno is recognized as an expert in myocardial protection through his innovative research in the laboratory and clinically. His published textbook is a classic on this topic and summarizes the current knowledge on all aspects of myocardial protection, including the latest information in the treatment of cardiac diseases, robotics, pediatric surgery, and treatment of cardiac failure.

Drs. Salerno, Buckberg, and Deslauriers worked together collaborating in basic and clinical research on myocardial protection. And Drs. Buckberg and Salerno submitted an abstract to the American Association for Thoracic Surgery based on their work on simultaneous antegrade/retrograde cardioplegia to optimize cardioplegic delivery^[3]^.

### Off-pump Coronary Artery Bypass Surgery

**Fig. 12:**
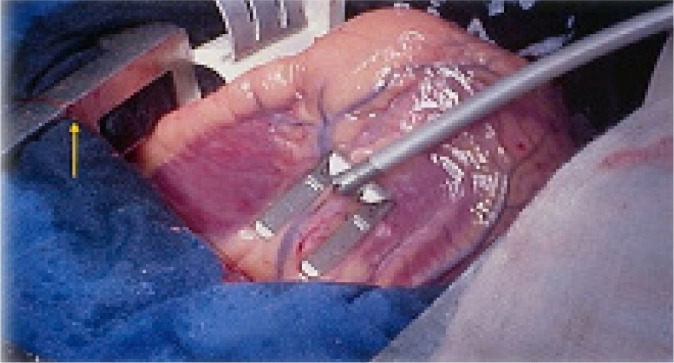
Lima suture developed by Dr. Ricardo Lima allowing to perform anastomoses on the posterior wall of the heart and complete revascularization.

The pioneering work of Enio Buffolo and Federico Benetti led Dr. Salerno to establish relationships with them, and these authors introduce in a major way the concept of off-pump coronary artery surgery. This technique avoids the use of the heart-lung machine and some of the sequelae related to brain injury, inflammatory response, etc. Dr. Salerno organized worldwide conferences on this technique, performed live demonstrations, and used the Lima suture (developed by Ricardo Lima, Brazil) (Figure 12)^[4]^, which allowed him to perform anastomosis in the posterior arteries of the heart. The repercussion of the technique was considerable because it solved the criticisms regarding incomplete revascularization in the off-pump coronary artery bypass grafting. Coronary surgery without the use of extracorporeal circulation has earned its place in the world of cardiac surgery. Dr. Salerno improved the Lima suture technique during this period^[5]^. And he became a national and international sought “inspirational” speaker on this topic of his passion. As a result, Dr. Salerno participated in the development of new technology and techniques, like the transit-time intraoperative blood flow meter that allowed him to confirm patency of the anastomosis performed, and developed and patented innovative products, such as the blower mister, without which this procedure cannot be done.

### Beating Heart Valve Surgery

Dr. Salerno developed innovative techniques in the areas of mitral valve, aortic valve, and aneurysms, etc. The benefits were better heart function, earlier recovery, and overall benefit for the patient. His work led to the realization of the need for delivery of oxygen to the heart, rather than relying on hypothermia for heart protection^[6]^.

### The Batista Operation (Partial Left Ventriculectomy)

Salerno introduced Dr. Randas Batista to the world once he realized the importance of this procedure in the treatment of congestive heart failure and dilated cardiomyopathy. Together they travelled all over the world, providing live demonstrations. This procedure established the basis for an interest in surgery treatment of dilated cardiomyopathy, avoiding heart transplantation, and allowing other surgeons to develop innovative new procedures^[7]^. The Batista operation seems to be an attractive choice to treat end-stage heart failure in Japan, where the option of treatment for this situation is limited.

### Pulmonary Protection During Heart Surgery

The lung is the “target organ”, the one that suffers most from ischemic injury during heart surgery, and there is no perfusion to the pulmonary artery, the lungs are not ventilated, and nutritive flow is based on the bronchial arteries, which now has no pulsatility and low pressure. Drs. Edmo Atique Gabriel, Enio Buffolo, and Tomas Salerno worked together in innovative ways of ventilating the lungs and at the same time perfusing the lungs as a mean of preventing ischemic injury^[8]^. Again, his textbook is a classic on this topic.

## ACCOMPLISHMENTS AND AWARDS

The Professor Tomas A. Salerno is an Honorary Professor of Cardiac Surgery at the University of Chieti (Italy) and has been recognized with the Best Doctors in America Awards. During more than 40 years of professional life, he has accumulated many awards and honors, for example:

1. 1998, Lister Prize of the University of Toronto.
2. 1999, Laurea Honoris Causa from Italy.
3. 2002, Laurea Honoris Causa from Argentina.
4. 2004, Montevergine Innovation Prize, Italy.
5. 2009, Salerno Miami Day Proclamation (May 7th), awarded by the Mayor of Miami Dade.
6. 2015, International Member of the National Academy of Medicine in Brazil.
7. 2016, Dewitt C. Daughtry Endowed Chair of Cardiothoracic Surgery of the University of Miami.
8. 2016, founding President of the Latin American Association of Cardiovascular and Thoracic Surgery.
9. 2020, Elected Member Iron Arrow, the highest honor of the University of Miami. The Iron Arrow Honor Society, founded in 1926, in conjunction with the University’s opening, is the Highest Honor Attained at the University of Miami. Based on Seminole Indian tradition, Iron Arrow recognizes those individuals in the University of Miami community who exemplify the five qualities of Iron Arrow: *“Love of Alma Mater, Character, Leadership, Scholarship, and Humility”* (Figure 13).
  **Fig. 13:**
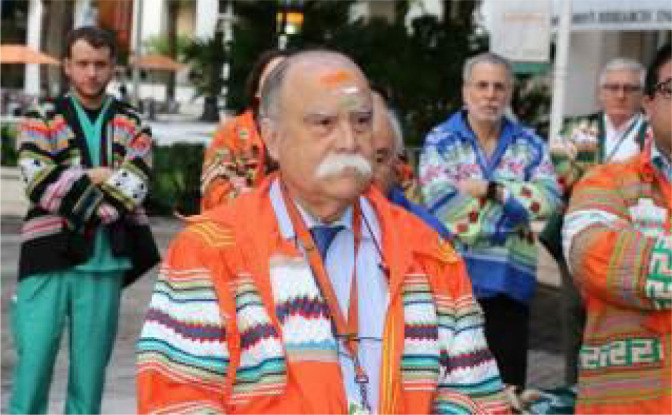
Prof. Salerno was elected Member Iron Arrow, the highest honor of Miami University in 2017.
10. 2021, Founding member of the Brazilian Academy of Cardiovascular Surgery.
11. 2021, Recipient of the International Coronary Congress (ICC) Lifetime Achievement Award for his contributions to the field of surgical myocardial revascularization. While Dr. Salerno has received numerous international accolades and awards, he acknowledges the ICC Lifetime Achievement Award as the most special, as it represents recognition by his peers worldwide.
12. 2022, Awarded the title of “Notório Saber” of the Universidade Federal do Ceará (Brazil), which can be requested by those who have high qualifications, demonstrated by experience and performance, that places them in intellectual prominence in the country, in his area of knowledge, and who have carried out a work recognized as relevant to the knowledge.

## PERSONAL LIFE

**Fig. 14:**
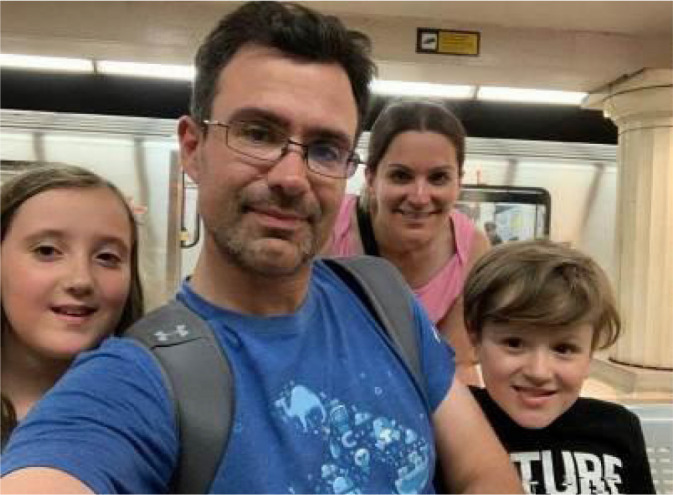
Tomas Salerno’s son Mark, grandchildren Amara and Riley, and daughter Kim.

**Fig. 15:**
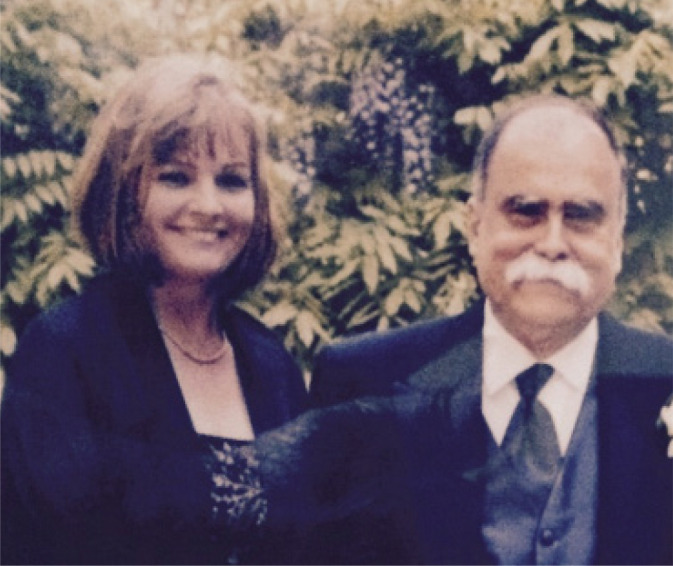
Helena and Tomas Salerno enjoying life during social event. They make a wonderful and charming couple.

Tomas A. Salerno has two children and two grandchildren (Figure 14). His son, Mark Salerno, is a businessman, and his daughter, Kim Salerno, works at Air Canada as a customer service agent. He married his second wife, Helena Salerno (Figure 15), and is now in a very loving relationship, with great respect and admiration. More than 10 years ago, Helena gave a great demonstration of her love for him by donating her kidney to Tomas at a difficult time in his life, suffering from terminal kidney failure.

## LEGACY

In addition to impeccable academic life, with his relevant research and experience including 41 published books/book chapters, over 500 authored peer-reviewed articles, member of 11 editorial boards of scientific journals, and over 292 national and international presentations, Dr. Salerno has made many other significant contributions in the development of cardiac surgery. He was responsible for bringing the Batista operation to North America, and Buffalo was the first center to perform this operation outside Brazil. This represents an important development in the treatment of end-stage heart diseases. This operation was performed all over the world, and the Cleveland Clinic held an important symposium paying attention to the possibility of Batista operation replacing heart transplantation. The treatment of end-stage heart failure was seen in a different light after the contribution of Drs. Batista and Salerno.

The Cardiac Center for Less Invasive Coronary Artery Surgery (Buffalo, New York) made a great contribution in development off-pump surgery (heart surgery without the heart-lung machine) in different ways, through small incisions (left anterior small thoracotomy) or via sternotomy, allowing total revascularization of the coronary arteries, including the posterior arteries of the heart. Near 95% of Salerno’s patients are operated upon without the heart-lung machine, receiving complete revascularization. With the collaboration of Drs. Enio Buffolo, Federico Benetti, Antonio Calafiore, Ricardo Lima, and others, he popularized the off-pump coronary artery bypass grafting all around the world by holding meetings, performing live surgeries, and receiving hundreds of visiting surgeons interested in learning the different techniques. This represented an important development in heart surgery.

As a result of years of experience working on myocardial protection, having contributed to warm blood cardioplegia development and introduced new concepts such as continuous retrograde warm blood cardioplegia, Dr. Salerno has been involved in heart valve procedures with the heart beating, utilizing antegrade and retrograde perfusion, without the use of potassium in cardioplegic solution. The benefit of this technique is that the heart is perfused throughout the aortic clamping period, thereby eliminating ischemia-reperfusion injury, allowing the operation of over 400 major valvular and other combined procedures.

Finally, Dr. Salerno is currently the editor-in-chief of the Journal of Cardiac Surgery, a leading journal in the field. He is also Chief Emeritus of Cardiothoracic Surgery at the Miller School and Jackson Memorial Hospital (the Jackson Health System made a difference in the lives of their patients, with a culture of innovation and exceptional care). And he is a professor at an important USA institution with more than 9,000 professionals, has trained more than 120 prominent national and international heart surgeons, and continues to influence students, residents, and surgeons as they pursue a career in cardiac surgery.

## CONCLUSION

These words summarize the importance of Tomas A. Salerno and his contributions during his professional/clinical career and his professor career serving the Academy. He is recognized by the worldwide cardiovascular surgery community in many areas, such as research, with a great creative potential. However, the most impressive fact is that Tomas is a very humble man and one of the most incredibly talented persons ever seen. When people meet him, they do not realize how important and recognized he is by his peers. Tomas gives recognition to others, and his life has touched many patients, families, physicians, colleagues, fellows, students, residents, nurses, and workers at his place of work. I venture to say that there is not a single cardiac surgeon in the world who has not heard of Dr. Tomas A. Salerno’s work, his contributions in the field of cardiac surgery, his clinical ability, and the impact that his research has had in changing how heart surgery is performed to better patient outcomes.

At the Miller School and the Jackson Memorial Hospital, Dr. Salerno held various chair positions, served as Chair of the Faculty Senate for the University of Miami, and was a member of many committees. This was all done while he continued his academic career in teaching, researching, and doing clinical work. He continues to be sought as a motivator and inspiring speaker in the field of cardiac surgery. He is considered a workaholic, but he enjoys it. The work is part of the joy of his life. How can a man produce so much clinical work, so much teaching, research, and service?

*“The only way to predict the future is to invent it yourself*”

Norman E. Shumway, Jr

## TESTIMONIALS

“Professor Tomas A. Salerno is an international leader in cardiac surgery and influenced generations of cardiac surgeons. His work spans basic science, clinical research, and innovations in cardiac surgery. He has been a mentor, and generous friend, to trainees. He is forward looking, always building our specialty. His greatest legacy is the lives he has touched. He continues to inspire all of us who have been fortunate to work with him.” (Antony Panos, MD)

“I have had the pleasure and privilege of having known Dr. Salerno for almost 3 decades. I have always been astonished by the wealth and extent of both his academic and clinical contributions to many areas of cardiothoracic surgery and particularly as one of the pioneers of off-pump CABG. Indeed, he was recently awarded the Lifetime Achievement of the 7th International Coronary Congress in 2021. And he continues to demonstrate great integrity in all that he does and is still accompanied by a great personal warmth and infectious sense of humor.” (David Target, MD)

“It is very easy to talk about Salerno: He is an illustrious Brazilian who has achieved success in Canada and the USA. The best qualification I can give to him is: he was an ambassador of Brazilian Heart Surgery in the USA, putting Brazilian surgeons and Brazilian Heart Surgery in all scientific forums. He wrote an editorial entitled *“Why Brazilian Heart Surgery is so Creative?”.* This was published and proves all its recognition for Brazilian Heart Surgery. He never omitted to be Brazilian and to represent Brazil abroad. Tomas Salerno is worthy of many tributes.” (Enio Buffolo, MD, PhD)

“Tomas A. Salerno is recognized worldwide as one of the most distinguished cardiovascular surgeons of his generation. This is due to his enormous contributions to the specialty, demonstrated in more than 500 published articles. Despite being an exponent in the field of cardiovascular surgery, he was always a great and fraternal friend of Brazilian surgeons, whom he always treated very closely and cordially, opening the doors of the important Cardiac Units that he led to many of us. With his prestige, he defended on the international scene many of the initiatives of Brazilian Cardiovascular Surgery, demonstrating his enormous appreciation for his origins.” (Fabio Jatene)

“Tomas Salerno made innumerable contributions to Cardiac Surgery. The current myocardial protection as well as coronary surgery without extracorporeal circulation - with his idea of the blower, among others - owes him recognition, but without a doubt, the most important thing about Tom is his greatness as a person and his selfless help and permanent pursuit of improving Cardiac Surgery.” (Federico Benetti, MD, PhD)

“A man is worth what he does during his life. To grow, it turns difficulties into opportunities. Open paths and share success with his peers. As a professor and head of service for competence and robust curriculum, he worked at three universities in North America, where he trained hundreds of surgeons. Generous, he opened space and hosted colleagues from Brazil. I did an internship with him in 1990 at St Michael’s Hospital, in Toronto, and in 1999 at Buffalo General Hospital, in Buffalo, NY. This is the remarkable surgeon/professor, Tomas A. Salerno, my unforgettable friend.” (Gilberto Barbosa, MD, PhD)

“In 1995, on a raining miserable evening in Warsaw after a meeting of the Polish national cardiac society, I came across this guy I had heard before but did not personally know, Tomas Salerno. He was playing on a slot machine with a bucket full of useless Polish coins in the hotel casino. Since there was not much else to do, I joined him, and it turned out to be the beginning of a long and productive friendship.” (G. D. Angelini, MD, MCh, FRCS, FMedSci)

“He was born in Cássia, a small town, and left home as a teenager to win the world, but in fact, the world won Salerno. He remained true to himself, his identity, and his heart. His life will always be remembered for the countless doors he opened for young surgeons. Awarded internationally by several institutions, he is intensely active in the scientific edition, with around 500 published articles, innovating and teaching the new generations in the History of Medicine and Cardiac Surgery” (José Glauco Lobo Filho, MD)

“Tomas Salerno, an example of life. A life that must be known and I pretentiously can summarize in a few words: determination, resilience, and love for human being. An excellent teacher, a passionate person, an ambassador for good causes. A great friend.” (José Teles de Mendonça)

“Tomas Salerno is one of those figures that we cannot define. Born Brazilian, proud of his native land, he became a citizen of the world. Standing out among his peers for the deep knowledge he added to himself, he was not fascinated by science as an expression of greatness. His generosity and selflessness captivate us and make us consider ourselves his blood brothers. His passion for cardiovascular surgery and for the human being is well demonstrated in his attitudes. I have a privilege to be part of the “Salerno friend family” and to have a mirror to guide us. In a stormy time when “myths” are sought to guide us, we find a human being of the greatest, equal to Jesus Christ, the simplest human that ever existed. Everything you receive in honors is little. I am a simple devotee.” (José Wanderley Neto, MD)

“I was lucky when I met Professor Tomas Salerno, more than 30 years ago, during the various scientific meetings he attended in Brazil. I am always impressed by his innovative capacity and his ability to transmit knowledge. Associated with all this is his friendliness and simplicity. At the end of the 1990’s, we built a solid friendship from his interest for Off-Pump Coronary Surgery and the possibility of revascularization of the posterior arteries of the heart. Frequently he visits our cardiac surgery unit at Real Hospital Português, Recife, Brazil. Salerno is a captivating, simple, friendly person as well as a charitable doctor. He is a great cardiac surgeon, with great scientific knowledge. Glad to be able to say “I’m your friend”.” (Mozart Escobar)

“How to define Professor Tomas Salerno with few words? Determined, exceptional human being, excellent surgeon, innovator, professor, human resources trainer, scientist, editor, and award-winning doctor. I could define it with many other adjectives and all with great intensity. I had the opportunity to meet him and live with him during my time as a visiting professor at the University of Toronto and since then I have had the honor of enjoying his friendship and knowledge.” (Paulo Broffman, MD, PhD)

“Professor Tomas Salerno, a first-class human and professional! I met him 52 years ago, during the Brazilian Cardiology Congress, held in Curitiba, when I was given the task of taking care of the American guest Prof. Dwight Harken from Harvard University. They liked me and took me to do residency in surgery. There I met Dr. Lair Ribeiro, who was doing research with Peter Maroko at the Peter Bent Brigham Hospital. When I was in Toronto doing a residency in Cardiac Surgery, Lair Ribeiro came to visit me, and we went together visit Prof. Salerno in Ottawa. Since then, we have become very good friends. I could see how exceptional Salerno is. Always ready to help anyone looking for him. Always willing to teach whoever needed it. He has become one of my favorite idols. In the late 1980’s, at a Cardiology Congress in Porto Alegre, I presented the work on “Ventriculoplasty at heart failure”. Salerno was incredulous and came to Curitiba city for a week when we operated on several patients, and he was amazed at the results; from then on, we were partners in the dissemination of the technique in Brazil and the world. This disclosure is because he is a surgeon with international credibility. I am very grateful to him for being one of his friends.” (Randas Vilela Batista, MD)

“Tomas Salerno is such a special person; few words, as the described above in this paper by surgeons and great friends, are so inadequate to describe all that he has accomplished, and how much he has meant to so many generations of trainees and cardiac surgeons around the world. He has been a real mentor, and a real friend to so many of us. He is a true friend and human being” (Ricardo Lima, MD)
